# Disruption of JAK2 in Adipocytes Impairs Lipolysis and Improves Fatty Liver in Mice With Elevated GH

**DOI:** 10.1210/me.2013-1110

**Published:** 2013-06-19

**Authors:** Sarah M. Nordstrom, Jennifer L. Tran, Brandon C. Sos, Kay-Uwe Wagner, Ethan J. Weiss

**Affiliations:** Cardiovascular Research Institute (S.M.N., J.L.T., E.J.W.), University of California, San Francisco, San Francisco, California 94158; Biomedical Sciences Graduate Program (B.C.S.), University of California, San Diego, San Diego, California, 92093; and Eppley Institute for Research in Cancer and Allied Diseases (K.U.W.), University of Nebraska, Omaha, Nebraska 68198

## Abstract

Nonalcoholic fatty liver disease (NAFLD) is considered the hepatic expression of the metabolic syndrome, and its prevalence is increasing. The factors that influence the development of fatty liver and its progression to steatohepatitis and cirrhosis are not well understood. The pleiotropic hormone, GH, has been associated with an increased risk of NAFLD in humans and mice. GH is known to have diverse effects on lipid metabolism including decreasing body fat in vivo, presumably through stimulation of lipolysis via an undefined mechanism. Previously we described mice with hepatocyte-specific deletion of the GH signaling mediator, Janus kinase 2 (JAK2L). JAK2L animals have elevated serum GH, reduced body fat, high liver triglyceride content, and increased serum markers of hepatocyte injury (alanine transaminase and aspartate transaminase). We aimed to determine whether the elevation of GH in JAK2L mice contributed to fatty liver by promoting lipolysis directly in adipocytes. We generated mice with adipocyte-specific disruption of JAK2 (JAK2A) and found that GH resistance in adipocytes reduced lipolysis and increased body fat. JAK2A mice were then crossed to JAK2L mice, and the resultant JAK2L/A animals had increased body fat and decreased lipolysis, despite elevated circulating GH. Furthermore, the increased triglyceride content, serum alanine transaminase, and serum aspartate transaminase observed in JAK2L mice were nearly normalized with the additional disruption of JAK2 in adipocytes (JAK2L/A mice). Our results offer novel mechanistic insights into the long-recognized effects of GH on lipid flux and suggest that GH signaling may play an important regulatory role in the development of NAFLD.

Nonalcoholic fatty liver disease (NAFLD) is increasingly prevalent worldwide. Major risk factors include obesity and type 2 diabetes mellitus; NAFLD is considered to be the hepatic signature of the metabolic syndrome. The mechanisms that underlie the development of fatty liver (FL) and its progression to nonalcoholic steatohepatitis and cirrhosis are not well defined, but various cytokines and hormone signaling pathways have been implicated. GH excess, GH deficiency, and defects in the GH signaling pathway have all been associated with the development of FL in mice and humans (1–3).

GH is a pleiotropic hormone that has important influences on growth and metabolism.

At the cellular level, GH signals through the growth hormone receptor (GHR), which lacks intrinsic kinase activity. Instead, signaling downstream of GHR requires the receptor-associated Janus kinase 2 (JAK2). JAK2 most commonly activates the signal transducer and activator 5 which, in turn, directly regulates the transcription of GH-dependent genes, most notably *Igf1*. The vast majority of circulating IGF-I is synthesized in the liver and serves as a plasma biosensor for circulating GH (1, 4, 5).

GH is well recognized to decrease fat mass, ostensibly via stimulation of lipolysis in adipose tissue. GH-deficient and GH-resistant patients have increased fat mass, whereas patients with excess circulating GH have reduced fat mass (6–9). Mouse models of decreased GH secretion or impaired GH signaling show similar changes in body composition (10–13). Despite the strong in vivo associations of GH and adiposity, the precise mechanisms by which GH influences fat mass remain unclear. GH may signal directly to adipocytes to modulate the rate of lipolysis, decrease de novo lipogenesis, or both. Alternatively, GH could influence adipocytes indirectly via signaling in neighboring cells or through modulation of an additional endocrine factor (14).

Previously, we showed that mice with liver-specific disruption of the GH signaling mediator, JAK2 (JAK2L), develop severe FL and have reduced body fat. The accumulation of liver triglyceride (TG) in JAK2L mice is dependent on dysregulated GH secretion resulting from the loss of IGF-I–mediated feedback inhibition. Based on these findings, we hypothesized that high levels of circulating GH in JAK2L mice would increase GH-stimulated lipolysis in adipose tissue, mobilizing free fatty acids (FFAs) for eventual uptake in hepatocytes (4). To determine whether the development of FL in JAK2L mice was dependent on GH-stimulated lipolysis, we disrupted JAK2 specifically in adipocytes (JAK2A mice) and then crossed JAK2A with JAK2L mice to generate mice with compound disruption of JAK2 in both hepatocytes and adipocytes (JAK2L/A). Like JAK2L mice, JAK2L/A mice had high circulating GH levels. However, fat mass was increased, and there was a near-total reduction in the rate of adipose tissue lipolysis in JAK2L/A mice. Strikingly, the increased liver lipid content and serum markers of hepatocyte injury found in JAK2L mice were largely normalized in JAK2L/A mice. In summary, we demonstrated that GH signaling in adipocytes is necessary for GH-stimulated lipolysis in adipose tissue, in vivo. Furthermore, disruption of GH-stimulated lipolysis in adipose tissue can prevent hepatic TG accumulation and hepatocyte injury in predisposed mice. This work provides a new understanding of the lipolytic actions of GH and highlights GH as an important regulator of lipid flux.

## Materials and Methods

### Animals

Mouse care and use for these studies were approved by the University of California, San Francisco Institutional Animal Care and Use Committee. Mice were maintained on a 12-hour light/dark cycle and were fed PicoLab Mouse Diet 20 (5058*; LabDiet, St Louis, Missouri) ad libitum. Mice with *loxP* sites flanking the first exon of *Jak2* were described previously (15). Adipocyte-specific JAK2-deficient mice (JAK2A) were generated by mating floxed JAK2 mice (JAK2^Flox/Flox^) in a mixed (C57BL/6:129Sv) background to mice expressing Cre recombinase under the control of the adiponectin promoter (*Adipoq-Cre*^+/−^) in an inbred C57BL/6 background (16). Hepatocyte-specific JAK2-deficient mice (JAK2L) were generated by mating floxed JAK2 mice (JAK2^Flox/Flox^) in a mixed (C57BL/6:129Sv) background to mice expressing Cre recombinase under the control of the albumin promoter (*Alb-Cre*^+/−^) purchased from The Jackson Laboratory (Bar Harbor, Maine) (17). JAK2L/A mice were generated by crossing JAK2L (JAK2^Flox/Flox^*Alb-Cre*^+/−^) and JAK2A (JAK2^Flox/Flox^*Adipoq-Cre*^+/−^) mice to generate offspring of 4 genotypes: CON (JAK2^Flox/Flox^*Alb-Cre*^−/−^*Adipoq-Cre*^−/−^), JAK2L, JAK2A, and JAK2L/A (JAK2^Flox/Flox^*Alb-Cre*^+/−^*Adipoq-Cre*^+/−^). Mice were born in the expected ratios. All experiments were performed on male mice between 8 and 10 weeks of age.

### Primary adipocyte isolation

Adipocyte cells were isolated from freshly excised fat pads essentially as described previously (18). In brief, fat pads were minced and incubated for 1 hour at 37°C with 5 mg/mL type I collagenase (Worthington, Lakewood, New Jersey) in Krebs-Ringer-bicarbonate HEPES buffer containing 10 mM sodium bicarbonate, 30 mM HEPES, 200 nM adenosine, 2.5 mM glucose, and 1% fatty acid-free BSA, pH 7.4 (Sigma-Aldrich, St Louis, Missouri). The digestion mixture was then filtered through a 100-μm nylon strainer (BD Biosciences, Franklin Lakes, New Jersey), and adipocytes were separated by flotation. Cells were washed 3 times with warm Krebs-Ringer-bicarbonate HEPES buffer, allowing for sufficient cell separation between each wash.

### Gene expression

Real-time quantitative PCR was performed using TaqMan primer/probe sets (5′FAM/3′BHQ; Biosearch Technologies, Novato, California) that were designed using Primer Express software (Applied Biosystems, Carlsbad, California) (see Supplemental Table 1 published on The Endocrine Society's Journals Online web site at http://mend.endojournals.org). Total RNA was then isolated using the RNeasy Lipid Tissue Kit (QIAGEN, Valencia, California) for adipocytes and the RNeasy Tissue Kit (QIAGEN) for muscle and liver according to the manufacturer's instructions. First-strand cDNA synthesis was performed using iScript Supermix for real-time quantitative PCR (Bio-Rad, Hercules, California) and oligo(dT) primers. Real-time quantitative PCR reactions were performed in a 384-well format using a Kapa Probe Fast qPCR kit (Kapa Biosystems, Woburn, Massachusetts) and total reaction volumes of 10 μl on an ABI 7900HT system (Applied Biosystems). Absolute gene expression (gene copy number) was quantified with the method of Dolganov et al (19) using the control genes *Gapdh*, β*-actin*, and *Mrps9*.

### Serum and plasma measurements

Blood was drawn via a retro-orbital puncture with a 1.5-cm segment of uncoated glass microcapillary tube. For serum, blood was drawn into empty tubes and incubated overnight at 4°C. Serum was separated by double-spinning at 13 000 relative centrifugal force for 15 minutes. Mouse IGF-I (R&D Systems, Minneapolis, Minnesota), mouse GH (Millipore, Billerica, Massachusetts), total mouse adiponectin (ALPCO, Salem, New Hampshire), and mouse leptin (ALPCO) were measured using standard ELISA assays according to the manufacturer's instructions. Biomarkers for liver and kidney function were measured at the University of California, Davis Comparative Pathology Laboratory.

### Body composition

Body composition was determined by dual-energy x-ray absorptiometry (DEXA). Live animals with an average age of 49 days were anesthetized with isoflurane and scanned on the Lunar PIXImus densitometer (GE Medical Systems, Fairfield, Connecticut). Percent fat was determined by manually dividing fat grams by mouse weight. For measurement of fat pad mass, mice were anesthetized with isoflurane and killed via cervical dislocation. Epididymal and inguinal fat pads were immediately removed, and wet fat pad weights were recorded.

### Metabolic cages

The comprehensive laboratory animal monitoring system (CLAMS; Columbus Instruments, Columbus, Ohio) was used to measure food intake, movement, oxygen consumption, and the respiratory exchange ratio over 4 consecutive days. Mice were housed in individual cages for 3 days and then were allowed to acclimate in the recording chambers for 24 hours before the start of measurement. Mouse weights and body composition were determined immediately after the monitoring period. Body composition was determined by quantitative magnetic resonance (QMR) on the EchoMRI-3in1 body composition analyzer (EchoMRI, Houston, Texas). Fat and lean mass were determined by the system software. We reported unnormalized values for all measures along with measurements of weight, lean mass, and fat mass. For measures of energy intake and expenditure, representative light and dark cycles were selected, and the measurements for that time period are reported.

### Stable isotope labeling

Total TG and palmitate content and newly synthesized TG and palmitate levels were measured at the Case Mouse Metabolic Phenotyping Center. To enrich body water with approximately 4% ^2^H_2_O, an ip injection of labeled water (20 μl/g body weight of 9 g/L NaCl in 99 atom percent excess ^2^H_2_O) was administered to 12-week-old male mice. Mice were returned to their cages for 7 days and allowed ad libitum access to food and 5% ^2^H-labeled drinking water. At sacrifice, blood and subcutaneous fat pads were collected, flash-frozen in liquid nitrogen, and stored at −80°C until analysis. TG concentrations and de novo lipogenesis were determined as described previously (20, 21). In brief, total TG and palmitate were isolated from subcutaneous fat pads using chemical hydrolysis and extraction techniques. The ^2^H-labeled glycerol and palmitate were analyzed after derivatization by mass spectrometry. The ^2^H-labeled TG covalently linked to glycerol measures the amount of newly synthesized TG, whereas the ^2^H-labeled TG covalently attached to palmitate indicates the amount of new palmitate. In mice given ^2^H_2_O for 7 days, the contribution of de novo lipogenesis to the total pool of TG and palmitate was calculated using the following equation: percent newly made palmitate = (total ^2^H-labeled palmitate · [^2^H-labeled body water × *n*]^−1^) × 100, where *n* is the number of exchangeable hydrogens, assumed to be 22 (20, 21). The percentage of total newly made TG-glycerol was calculated using the following formula: percent total newly made TG-glycerol = (^2^H-labeled TG-glycerol · [^2^H-labeled water × *n*]^−1^) × 100, where ^2^H-labeled TG-glycerol is the M1 isotopomer, ^2^H-labeled water is the average amount labeled in a given mouse, and *n* is the exchange factor (experimentally determined from the M2/M1 ratio of TG-glycerol). Net lipolysis was estimated from the direct measure of fractional TG synthesis and the change in body fat content during the label period with the following formula: net lipolysis = [percent total newly made TG-glycerol × (adipose tissue mass/labeling time) − (Δ adipose tissue mass/labeling time)]/fat pad mass.

### Liver triglyceride content

A portion of liver from each mouse was homogenized in buffer A (250 mM sucrose and 50 mM Tris, pH 7.4) at a concentration of 50 mg of tissue per 1 ml of buffer. Lysate was then added to Infinity Triglyceride Reagent (Thermo Scientific, Waltham, Massachusetts) to determine TG content, according to the manufacturer's instructions.

### Liver histology

Mice were anesthetized with isoflurane and sacrificed via cervical dislocation. Liver tissue was immediately excised and incubated overnight in PBS containing 10% sucrose. Tissue was then embedded in O.C.T. Compound (Tissue-Tek; Sakura Finetek, Torrance, California) on dry ice and placed at −80°C until processing. Sectioning and hematoxylin and eosin staining were performed at the University of California, San Francisco Helen Diller Cancer Center Mouse Pathology Core.

### Statistics

A Student *t* test was used to determine significance when 2 groups were compared. For comparison of 3 or more groups, a 1-way ANOVA was used, followed by the Bonferroni posttest with selected comparisons. Analysis of 2 or more groups over time was performed using a 2-way ANOVA followed by the Bonferroni posttest. An α value of .05 was set for all statistical tests. Data are presented as means ± SEM. All statistical analyses were performed with GraphPad Prism statistical software (version 5; GraphPad Software, La Jolla, California).

## Results

### Cell-specific deletion of JAK2 disrupts GH signaling in target tissues

**Figure 1.**
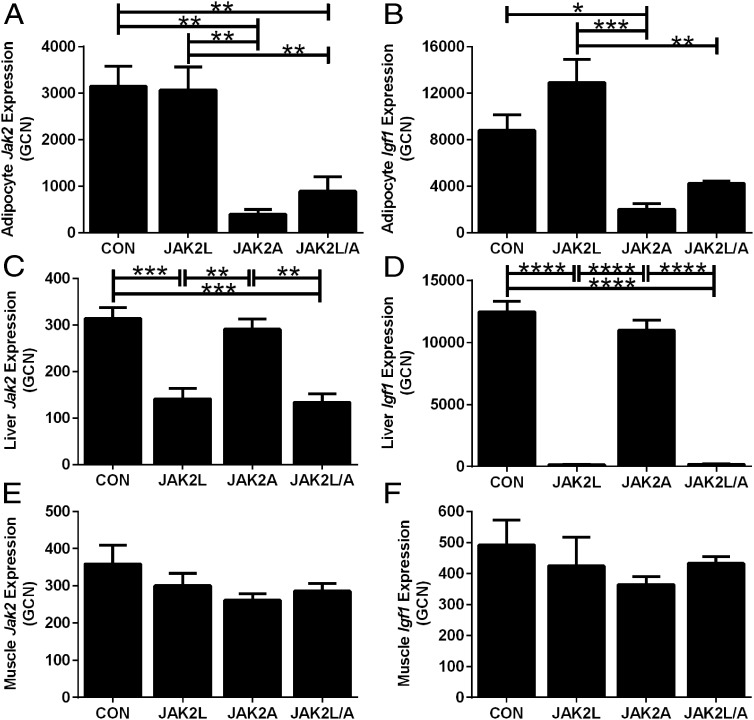
Tissue specificity of *Jak2* deletion and disrupted GH signaling. JAK2A mice were crossed to JAK2L mice, generating JAK2L/A animals that are doubly deficient of *Jak2* in hepatocytes and adipocytes. Primary adipocytes, liver tissue, and muscle tissue were isolated from CON, JAK2L, JAK2A, and JAK2L/A animals. From these samples, we assessed expression levels of *Jak2* to confirm the degree and specificity of gene deletion. We also measured expression levels of *Igf1* as an indicator of intact GH signaling. A and B, Absolute gene expression of *Jak2* and *Igf1* was decreased in JAK2A and JAK2L/A adipocytes. JAK2L mice had similar expression levels of *Jak2* in adipocytes and showed a trend toward increased *Igf1* expression vs CON, in concordance with high circulating GH (n = 4–6). C and D, JAK2L and JAK2L/A mice had decreased expression of *Jak2* and *Igf1* in liver tissue, whereas JAK2A livers had expression comparable to that of CON livers for both genes (n = 4–6). E and F, Expression of *Jak2* and *Igf1* in muscle tissue was comparable among all 4 genotypes (n = 4–5). Data are expressed as means ± SEM. *, *P* < .05; **, *P* < .01; ***, *P* < .001; ****, *P* < .0001.

Mice with adipocyte-specific deletion of *Jak2* (JAK2A) were viable and had no gross abnormalities compared with their littermate controls (CON). JAK2A mice were crossed to previously described JAK2L animals to generate mice with combined deficiency of both hepatocyte and adipocyte *Jak2*. The breeding strategy was designed to yield offspring of 4 genotypes: CON, JAK2L, JAK2A, and JAK2L/A. Mice were born in the expected ratios. To confirm deletion of *Jak2* and disruption of GH signaling in the appropriate tissues of all 4 genotypes, we assessed gene expression from primary adipocytes, liver tissue, and muscle tissue using specific TaqMan primer probe sets validated for each gene (Supplemental Table 1). We measured gene expression of *Jak2* to confirm the primary deletion and *Igf1* as an indicator of intact GH signaling. Expression of *Jak2* in adipocytes was significantly decreased in both JAK2A and JAK2L/A compared with that in CON and JAK2L adipocytes (Figure 1A; n = 5–6, *P* < .01). Likewise, *Igf1* expression was significantly decreased in JAK2A and JAK2L/A adipocytes compared with that in CON and JAK2L adipocytes, respectively (Figure 1B; n = 5–6, *P* < .05 and *P* < .01, respectively). Deletion of *Jak2* in hepatocytes resulted in a significant reduction of *Jak2* expression in JAK2L (55%) and JAK2L/A liver tissue (57%) compared with that in CON liver tissue (Figure 1C; n = 5–6, *P* < .001), whereas JAK2A liver gene expression was normal. Expression of *Igf1* was 99% reduced in both JAK2L and JAK2L/A liver tissue compared with that in CON liver tissue (Figure 1D; n = 5–6, *P* < .0001), whereas JAK2A *Igf1* liver gene expression was normal. Muscle gene expression of both *Jak2* and *Igf1* was comparable among all genotypes (Figure 1, E and F; n = 5–6, not significant [NS]).

**Figure 2.**
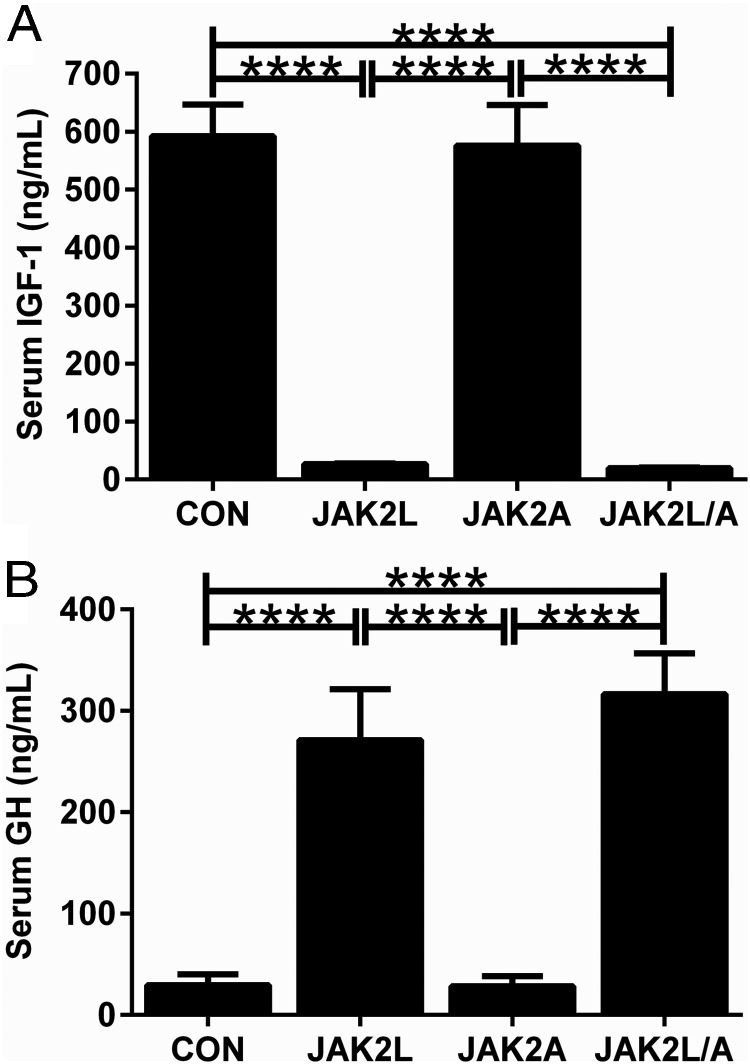
Hepatocyte-specific deletion of *Jak2* leads to large reductions in circulating IGF-I and increased circulating GH. There was a significant increase in circulating GH (A) and a decrease in IGF-I (B) in JAK2L and JAK2L/A mice, but there were no changes in either GH or IGF-I in JAK2A mice (n = 8).

In congruence with the dramatic reduction in liver *Igf1* expression and the fact that the vast majority of circulating IGF-I is synthesized by the liver, JAK2L and JAK2L/A mice had a >95% reduction in circulating IGF-I compared with that for CON mice (Figure 2A; n = 6–9, *P* < .001). Circulating IGF-I normally acts as a feedback inhibitor of GH secretion from the anterior pituitary; there was a 9-fold increase in circulating GH levels in JAK2L and JAK2L/A mice compared with that in CON mice (Figure 2B; n = 7–8, *P* < .001).

### Mice with adipocyte-specific disruption of JAK2 have increased body fat

**Figure 3.**
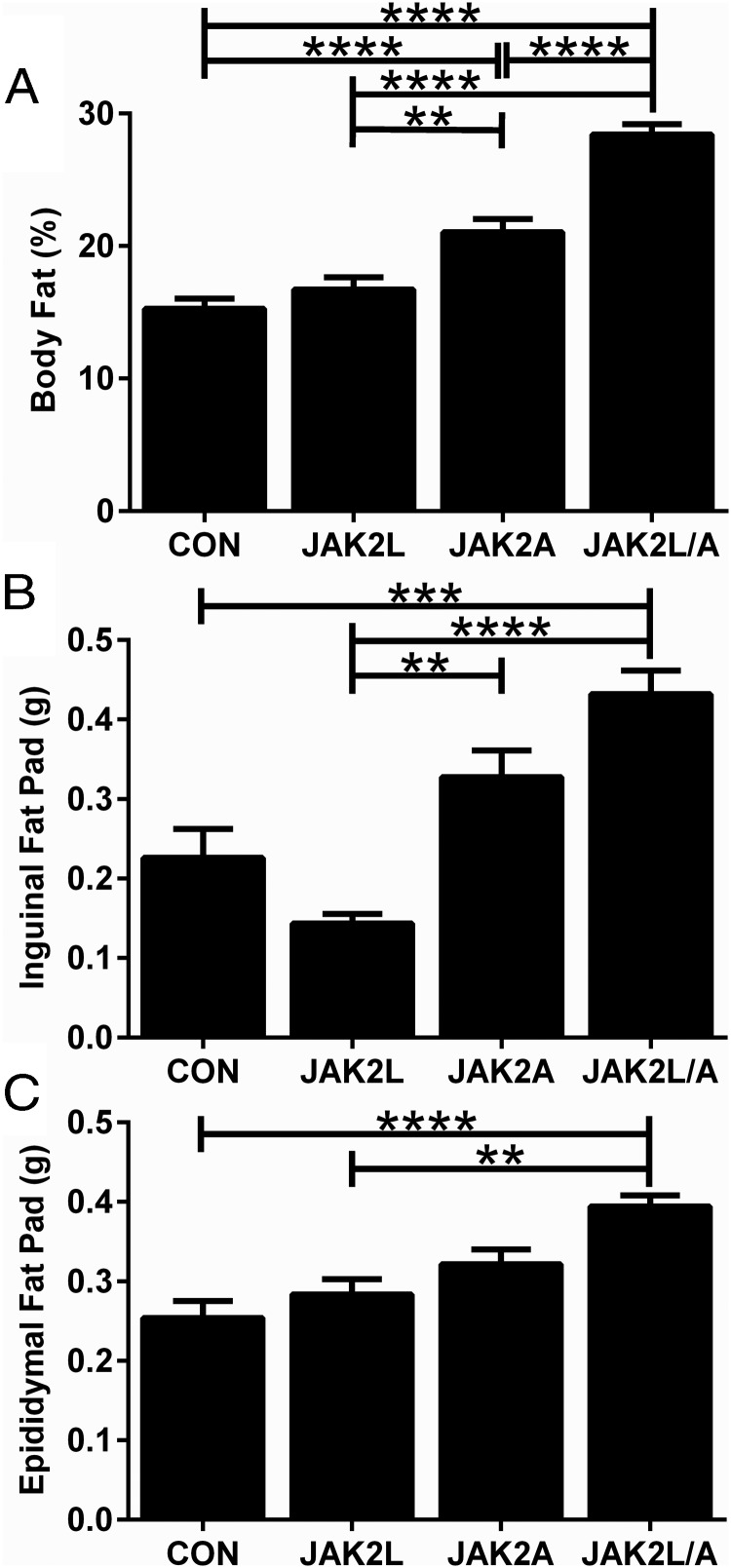
Mice with adipocyte-specific disruption of JAK2 have increased body fat. A, JAK2A and JAK2L/A mice had increased percent body fat vs CON mice as measured by DEXA. There were no differences between JAK2L and CON mice (n = 14–20). B, Inguinal fat pad weights were nonsignificantly decreased in JAK2L mice and nonsignificantly increased in JAK2A mice vs CON mice. JAK2L/A mice had significantly greater inguinal fat pad weights vs CON mice (n = 5–7). C, Epididymal fat pad weights of JAK2L mice were similar to those of CON mice. JAK2A mice had a nonsignificant increase in epididymal fat, whereas JAK2L/A mice had a significant increase vs CON mice (n = 10–12). All data are expressed as means ± SEM. **, *P* < .01; ***, *P* < .001; ****, *P* < .0001.

Given the long-standing interest in the effects of GH on adiposity and the presumption of decreased lipolysis in mice with disrupted GH signaling in adipocytes, we used DEXA to determine the body composition of CON, JAK2L, JAK2A, and JAK2L/A mice. There were no significant differences in percent body fat between JAK2L and CON mice (Figure 3A; n = 17–20, NS), despite the increased circulating GH levels in JAK2L mice. This probably resulted from an overestimation of body fat content by DEXA due to the high lipid content of the liver in JAK2L mice (4). In contrast, there was a 38% increase in body fat in JAK2A vs CON mice (Figure 3A, n = 18–20, *P* < .001) and an 86% increase in body fat in JAK2L/A vs CON mice (Figure 3A, n = 14, *P* < .001). We found similar changes in body composition using QMR (Table 1).

**Table 1.** Metabolic and Body Composition Parameters

	CON		JAK2L		JAK2A		JAK2L/A	
	Light	Dark	Light	Dark	Light	Dark	Light	Dark
Total food intake, g	1.44 ± 0.27	2.95 ± 0.14	1.42 ± 0.06	2.19 ± 0.29	1.31 ± 0.09	2.56 ± 0.29	1.12 ± 0.15	2.65 ± 0.19
Average O_2_ volume, L	125 611 ± 5459	137 735 ± 6475	106 344 ± 5591	117 835 ± 8450	109 980 ± 5449	124 042 ± 5815	104 073 ± 2236	113 659 ± 2178
Average respiratory exchange ratio	0.89 ± 0.02	0.93 ± 0.02	0.87 ± 0.02	0.92 ± 0.02	0.90 ± 0.01	0.95 ± 0.02	0.88 ± 0.01	0.94 ± 0.01
Total × movement, counts	2640 ± 153	5552 ± 314	2834 ± 110	5872 ± 376	2586 ± 317	6051 ± 827	2787 ± 148	5123 ± 187
Weight, g	27.67 ± 0.60		22.17 ± 0.53^a^		27.78 ± 0.41		24.57 ± 0.94^b^	
Lean mass, g	23.12 ± 0.25		18.65 ± 0.43^a^		21.63 ± 0.47		17.27 ± 0.51^a^	
Fat mass, g	3.75 ± 0.54		3.17 ± 0.31		5.38 ± 0.43		6.94 ± 0.44^a^	

Energy intake and expenditure was assessed using the CLAMS system immediately followed by QMR scanning for body composition. Representative light and dark cycles were selected from the measurement period, and raw values are expressed as means ± SEM; n = 6.

a *P* < .0001 relative to CON.

b *P* < .05 relative to CON.

We isolated fat pads of mice and found a nonsignificant reduction in inguinal fat pad mass in JAK2L vs CON mice (Figure 3B, n = 6, NS), consistent with increased circulating GH. In contrast, there was a nonsignificant 45% increase in inguinal fat pad mass in JAK2A mice (Figure 3B; n = 7, NS) and a 91% increase in JAK2L/A mice (Figure 3B, n = 6, *P* < .001) compared with that in CON mice. There was no difference in epididymal fat pad mass between JAK2L and CON mice, despite increased GH. There was also no difference in epididymal fat pad mass between JAK2A and CON mice, but there was a significant increase in the epididymal fat pad mass of JAK2L/A compared with that in CON mice (Figure 3C, n = 10–13, *P* < .0001).

**Figure 4.**
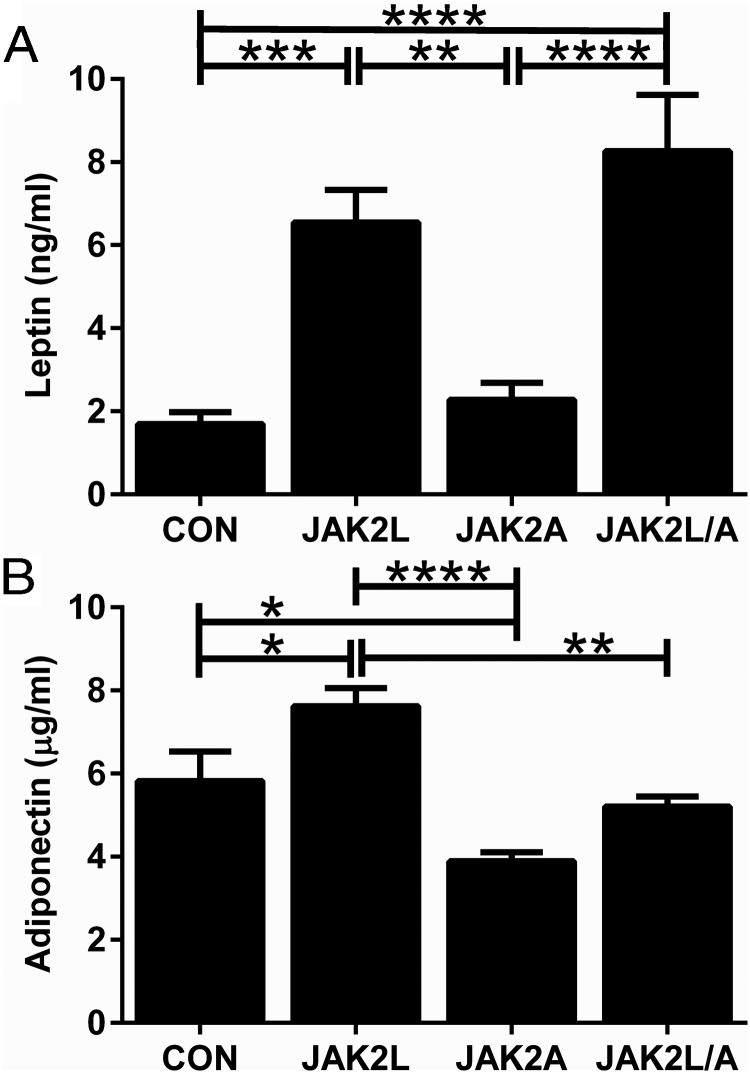
Mice with hepatocyte and/or adipocyte specific deletion of JAK2 have altered serum adipokines independent of adiposity. A, JAK2L and JAK2L/A mice had significantly increased circulating leptin compared with that in CON and JAK2A mice (n = 14–21). B, JAK2L mice had significantly increased circulating adiponectin, whereas JAK2A mice had reduced circulating levels compared with those in CON mice (n = 6–9).

We found marked changes in both serum leptin and adiponectin levels. Leptin was increased in JAK2L mice (Figure 4A, n = 14–21, *P* < .001) and in JAK2L/A vs CON mice (Figure 4A, n = 14–21, *P* < .0001). There were comparable levels of circulating leptin in JAK2A and CON animals (Figure 4A, n = 14–21, ns). There were also significant differences in total adiponectin levels: JAK2L mice had significantly higher levels of circulating adiponectin compared with those in CON mice (Figure 4B, n = 6–9, *P* < .05), whereas JAK2A mice had significantly lower adiponectin vs CON mice (Figure 4B, n = 6–9, *P* < .05). There was no difference in adiponectin between JAK2L/A and CON mice (Figure 4B, n = 6–9, NS).

### Mice with adipocyte-specific disruption of JAK2 have reduced lipolysis in vivo

Thus far, we showed that deletion of JAK2 in adipocytes ablates GH signaling and leads to increased body fat. As discussed previously, GH has been assumed to promote lipolysis; however, the observed changes in body composition could also theoretically result from changes in the rate of lipolysis, lipogenesis, energy intake, energy expenditure, or a combination of these factors.

To assess the mechanism underlying the change in body composition, we measured energy intake and expenditure via indirect calorimetry. There were no significant changes in food intake, activity levels, oxygen consumption, or the respiratory exchange ratio between genotypes, although CON mice did show a trend toward higher average oxygen consumption and food intake in the dark cycle (Table 1; n = 6, NS). Based on these findings, we concluded that the increased adiposity of JAK2A and JAK2L/A mice was not due to primary changes in energy intake or expenditure.

Previously, we observed a significant increase in circulating free fatty acids in JAKL vs CON mice (4). When JAK2L mice were crossed to GH-deficient *little* mice, the FFA levels were normalized. In the current study, plasma measurements of FFAs were also significantly increased in JAK2L compared with CON mice (Supplemental Figure 1; n = 7–11, *P* < .05; Supplemental Methods); FFA levels were completely normalized in JAK2L/A animals (Supplemental Figure 1; n = 7–11, ns).

**Figure 5.**
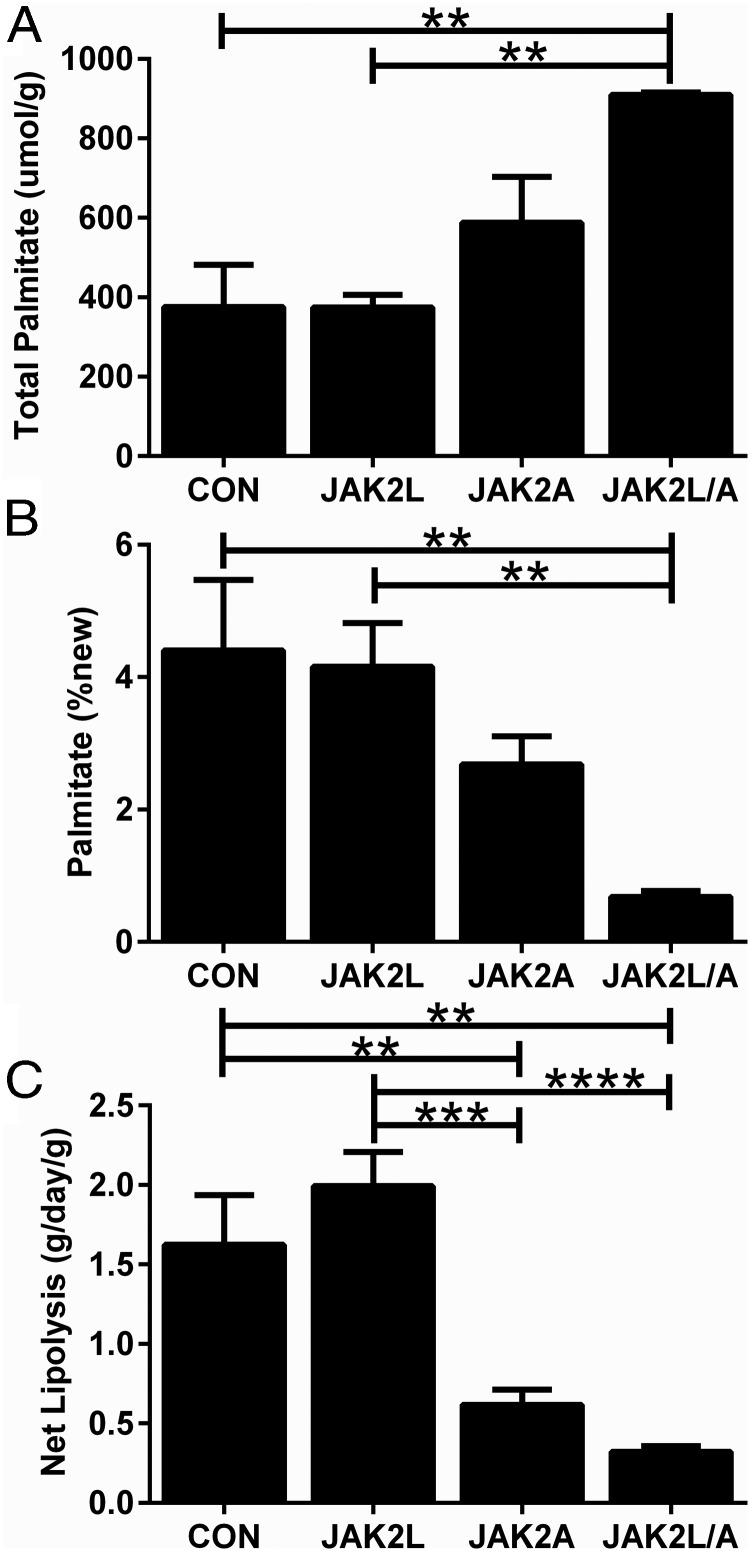
Mice with adipocyte-specific disruption of JAK2 have reduced lipolysis in vivo. De novo lipogenesis and net lipolysis were measured in white adipose tissue by stable isotope labeling of newly synthesized fatty acids and glycerol over a 7-day period. A, Total fatty acid content was nonsignificantly increased in JAK2A mice and significantly increased in JAK2L/A vs CON mice. B, Newly synthesized fatty acids were nonsignificantly decreased in JAK2A mice and significantly decreased in JAK2L/A vs CON mice. C, Net lipolysis was significantly decreased in JAK2A and JAK2L/A vs CON mice. All data are expressed as means ± SEM (n = 6). **, *P* < .01; ***, *P* < .001; ****, *P* < .0001.

Next, we sought to determine the effect of adipocyte-specific *Jak2* deletion on the rates of de novo lipogenesis and lipolysis in subcutaneous adipose tissue in vivo. We followed the previously described methods for stable isotope labeling of newly synthesized fatty acids and glycerol over a 7-day period (22). Consistent with increased inguinal fat mass, the total amount of palmitate in adipose tissue samples from the inguinal depot was nonsignificantly increased in JAK2A mice (Figure 5A; n = 5–6, NS) and significantly increased in JAK2L/A mice (Figure 5A; n = 5–6, *P* < .01) compared with that in CON mice. The amount of newly synthesized palmitate was nonsignificantly decreased in JAK2A mice (Figure 5B; n = 5–6, NS) and significantly decreased in JAK2L/A mice (Figure 5B; n = 5–6, *P* < .01) compared with that in CON mice. There were no significant differences between JAK2L and CON mice for either measure (Figure 5, A and B; n = 5–6, NS). Net lipolysis was estimated based on a previously validated formula that relates the direct measure of fractional TG synthesis to the change in body fat content during the label period (22). Net lipolysis was increased by 23% in JAK2L compared with CON mice (Figure 5C; n = 5–6, NS), consistent with the chronic elevation in circulating GH and resultant decrease in body fat measured in JAK2L mice. In contrast, net lipolysis was decreased by 62% in JAK2A mice (Figure 5C; n = 5–6, *P* < .01) and 80% in JAK2L/A mice (Figure 5C; n = 5–6, *P* < .01). Overall, these data suggest that the increased fat mass of mice with disrupted adipocyte GH signaling results from a decrease in the rate of lipolysis in vivo.

### Adipocyte-specific disruption of JAK2 largely rescues the fatty liver in JAK2L mice

Above we demonstrated that adipocyte-specific disruption of JAK2 resulted in a significant decrease in the rate of lipolysis in vivo. Previously, we showed that mice with hepatocyte-specific deletion of JAK2 (JAK2L) develop severe fatty liver, and the increased TG content of JAK2L livers is dependent on elevated circulating GH (4). We hypothesized that the increase in circulating GH promotes lipolysis directly in adipocytes, ultimately leading to increased hepatic uptake of FFAs and the development of fatty liver. To formally test the hypothesis that FL in JAK2L mice resulted from increased GH-stimulated lipolysis, we crossed JAK2L to JAK2A mice to generate mice with combined deficiency of JAK2 in both hepatocytes and adipocytes, referred to as JAK2L/A. Like JAK2L, JAK2L/A mice had increased circulating GH; however, unlike JAK2L, JAK2L/A mice were GH-resistant in adipocytes and had reduced net lipolysis. We sought to determine the significance of adipocyte GH signaling in the development of FL in JAK2L animals.

**Figure 6.**
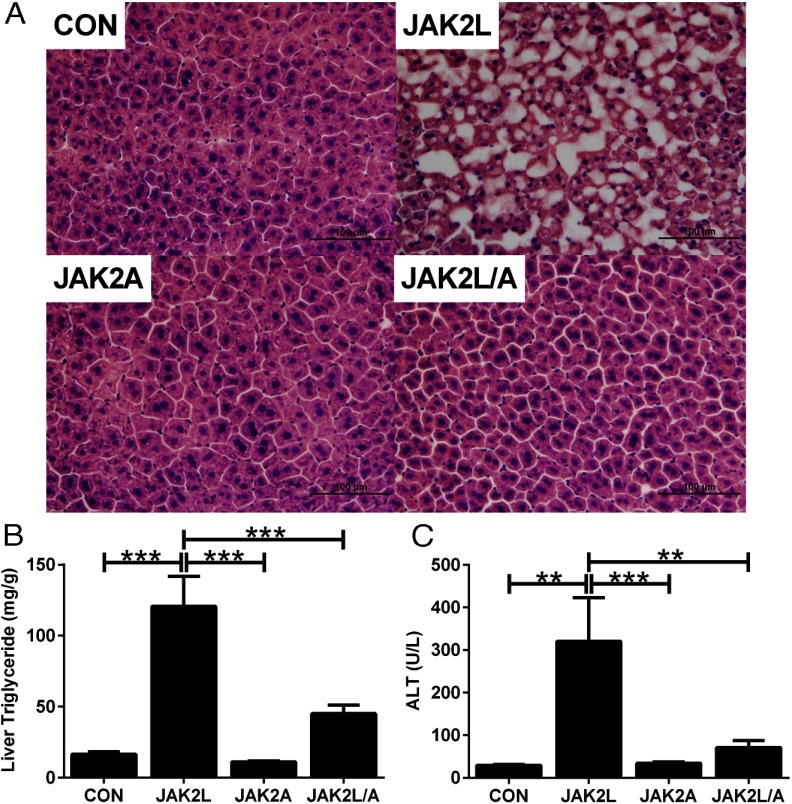
Adipocyte-specific disruption of JAK2 rescues the fatty liver in JAK2L mice. A, JAK2A mice were crossed to JAK2L mice to generate JAK2L/A animals that are doubly deficient in *Jak2* expression in hepatocytes and adipocytes. B, Liver triglyceride content was quantified from homogenized liver by a colorimetric assay. C, A marked increase in serum ALT was seen in JAK2L mice, whereas serum ALT content was nearly normalized in JAK2L/A mice (n = 10–14). All data are expressed as means ± SEM. **, *P* < .01; ***, *P* < .001.

In agreement with our previous findings, there was a profound increase in hepatocyte cytoplasmic vacuolization (Figure 6A) and a 7.3-fold increase in liver TG content in JAK2L vs CON mice (Figure 6B; n = 10–14, *P* < .0001). In contrast, the liver TG content of JAK2A and JAK2L/A mice was qualitatively (Figure 6A) and quantitatively (Figure 6B; n = 10–14, NS) comparable to that in CON mice. Furthermore, the level of serum alanine transaminase (ALT) was elevated in JAK2L compared with that in CON mice (Figure 6C and Table 2; n = 5–7, *P* < .01), indicating hepatocyte injury. In JAK2L/A animals, serum ALT was comparable with that in CON mice (Figure 6C and Table 2; n = 5–7, NS). Similar results were found with the measurement of serum aspartate transaminase (Table 2). These findings demonstrate that deletion of JAK2 in adipocytes was sufficient to prevent liver lipid accumulation, inflammation, and cell death in JAK2L mice; hence, disruption of GH signaling in adipocytes through the deletion of JAK2 inhibits the GH-dependent mobilization of FFA from adipose tissue, thereby preventing the development of fatty liver.

**Table 2.** Serum Biomarkers of Liver and Kidney Function

	CON	JAK2L	JAK2A	JAK2L/A
Albumin, g/dL	2.99 ± 0.05	3.30 ± 0.08^a^	2.87 ± 0.03^b^	3.35 ± 0.06^a,c^
Alkaline phosphatase, U/L	100.6 ± 8.2	100.9 ± 6.3	125.2 ± 6.3	117.1 ± 11.3
ALT, U/L	29.55 ± 2.05	320.00 ± 102.9^a^	34.37 ± 3.10^b^	70.97 ± 16.16^d^
Aspartate transaminase, U/L	56.62 ± 3.41	153.60 ± 22.58^e^	72.53 ± 4.82^d^	93.13 ± 15.45^f^
Blood urea nitrogen, mg/dL	33.08 ± 1.18	37.09 ± 0.84	34.17 ± 2.40	44.38 ± 2.69^a,g^
Creatinine, mg/dL	0.192 ± 0.027	0.203 ± 0.020	0.189 ± 0.017	0.219 ± 0.028
Total bilirubin, mg/dL	0.034 ± 0.002	0.063 ± 0.003^e^	0.038 ± 0.002^b^	0.045 ± 0.005^f^
Total protein, g/dL	4.43 ± 0.06	4.44 ± 0.02	4.21 ± 0.04^a,f^	4.32 ± 0.04

Values are expressed as means ± SEM; n = 6–7.

a *P* < .01 relative to CON.

b *P* < .001 relative to JAK2L.

c *P* < .001 relative to JAK2A.

d *P* < .01 relative to JAK2L.

e *P* < .001 relative to CON.

f *P* < .05 relative to JAK2L.

g *P* < .01 relative to JAK2A.

## Discussion

GH has a powerful effect on lipid metabolism in humans and in animal models. Previously, we showed that disruption of GH signaling in hepatocytes in JAK2L mice leads to a reduction in body fat and severe FL that is dependent on dysregulated GH secretion (4). In the present study, we showed that the loss of GH signaling specifically in adipocytes, via disruption of JAK2, led to (1) a significant reduction in the rate of adipose tissue lipolysis in vivo, (2) an increase in adiposity in vivo, particularly in the inguinal fat depot, and (3) prevention of liver TG accumulation and associated increases in markers of hepatocyte injury in JAK2L mice with high levels of circulating GH and disrupted hepatocyte GH signaling (4).

Mice with fat-specific deletion of GHR (FaGHRKO) were recently described, and there are important differences between this model and the adipocyte-specific disruption of JAK2 reported in this study. The most notable difference is a greater accumulation of fat in FaGHRKO mice than in JAK2A mice (23). In addition to potential differences associated with excising JAK2 vs GHR, this difference may also be attributed to variations in age, genetic background, or the Cre recombinase (*Cre*) promoters used to excise the relevant alleles in fat. The *Adipoq-Cre* line used in our model is selectively active in mature adipocytes of white and brown adipose tissue (16, 24) in contrast to the *FABP4/aP2* promoter of FaGHRKO mice, which has been shown to drive *Cre* expression in a variety of cells and tissues, including macrophages and skeletal muscle (23, 24). Considering that GH signals in many cell types and has diverse effects on metabolism, the differences in *Cre* selection are important to consider for this and future comparisons of these animals. In addition, although JAK2 is known to be an essential component in GH signaling downstream of GHR, JAK2 is involved in additional signaling pathways that could contribute to the observed phenotypes. Although we cannot formally rule out the possibility that deletion of JAK2 might affect pathways beyond GH signaling, we have previously reported that the hepatocyte-specific deletion of JAK2 (JAK2L) nearly phenocopies mice with hepatocyte-specific deletion of GHR (GHRLD). Despite all of the caveats described above, JAK2A mice and FaGHRKO mice demonstrate many similarities including the overall increase in adiposity and changes in levels of serum adipokines (4, 23). Ongoing and future studies will be needed to determine which, if any, additional JAK2-dependent signaling pathways are altered in JAK2A and JAK2L/A mice and whether these pathways significantly contribute to the observed phenotypes.

It is also important to note that there was a 9-fold increase in circulating GH levels in JAK2L and JAK2L/A mice compared with that in CON and JAK2A animals. This is due to the loss of IGF-I–mediated feedback inhibition on GH secretion and results in supraphysiologic levels of circulating GH. However, despite the large increase in circulating GH, the rate of net lipolysis was decreased, and the fat mass was increased in JAK2L/A mice.

We observed greater differences in inguinal fat than in epididymal fat in JAK2A and JAK2L/A mice. The enhanced response of the inguinal depot to disrupted GH signaling is not understood, although differential responses between these depots have been documented (12, 23). Inguinal fat has been shown to differ in matrix composition, lipolytic sensitivity, hormone receptor densities, and cellular composition compared with epididymal fat (25). Circulating leptin, which normally correlates positively with fat mass, was unchanged in JAK2A mice despite increased adiposity. Similar results were also noted in FaGHRKO mice. In contrast, we found increased leptin in lean JAK2L mice, as well as obese JAK2L/A mice. Leptin is reportedly elevated in GHR^−/−^ mice and in mice with liver-specific deletion of IGF-I (10, 26). We suggest that the increased circulating leptin in JAK2L and JAK2L/A mice may be due to reduced circulating IGF-I, as an inverse correlation between these factors was reported previously (27, 28). Like FaGHRKO mice, JAK2A animals had reduced total adiponectin; conversely, adiponectin was increased in JAK2L mice. This finding is in agreement with the reported negative correlation of serum adiponectin with obesity (29). To our surprise, adiponectin levels were not decreased in JAK2L/A mice despite obesity, indicating that another important regulatory component is involved.

In this study, we characterized 2 distinct mouse models with genetic disruption of GH signaling, JAK2A and JAK2L/A. In both models, JAK2 was deleted from adipocytes using the identical *Cre* promoter. It was, therefore, quite unexpected to find what appeared to be an exaggerated phenotype in JAK2L/A mice compared with that in JAK2A mice; specifically, JAK2L/A mice had greater fat mass than JAK2A mice by all measures. In addition, there was nearly a 50% decrease in the rate of lipolysis in JAK2L/A mice compared with that in JAK2A mice, in vivo. Discrepancies between JAK2A and JAK2L/A mice probably arise from the additional JAK2 disruption in hepatocytes of JAK2L/A mice and the resultant changes in circulating IGF-I, GH, and/or other hepatokines. Further studies are needed to assess this difference.

Overall, this study demonstrates a significant effect of GH on lipid homeostasis. To our knowledge, this is the first study to demonstrate the importance of adipocyte-specific GH signaling in the development of fatty liver. Our results also offer novel insights into the long-recognized effects of GH on lipolysis, demonstrating that GH signaling via JAK2 in adipocytes is important in fat metabolism in vivo. We have also shown that decreasing the rate of lipolysis led to a significant reduction in neutral lipid accumulation as well as serum markers of hepatocyte damage in JAK2L mice (4). This interesting observation suggests that the accumulation of neutral lipid in JAK2L mice is not entirely benign. In our model, FFAs are mobilized via effects of GH on adipocytes, taken up by hepatocytes, and reesterified. Despite the increased lipid content of JAK2L liver, we only observed a modest increase in hepatocyte inflammation. However, disruption of JAK2 in adipocytes conferred significant protection from hepatocyte injury as indicated by serum markers. This result has important implications for understanding the progression from benign hepatic steatosis to nonalcoholic steatohepatitis.

Finally, given the increased interest in the use of GH as a therapeutic agent to “treat” obesity (30) and the intense activity in the clinical development of JAK2 inhibitors for the treatment of cancer and inflammatory diseases, this work has significant translational implications (31, 32). Our work suggests that, although specifically antagonizing JAK2 or GH signalizing in hepatocytes might be expected to lead to FL, systemic antagonism might not. We hope that this and future work will aid in understanding the mechanisms underlying obesity and nonalcoholic fatty liver disease and potential insights into novel therapeutics for them.
